# The Anti-Allergic Rhinitis Effect of Traditional Chinese Medicine of Shenqi by Regulating Mast Cell Degranulation and Th1/Th2 Cytokine Balance

**DOI:** 10.3390/molecules22030504

**Published:** 2017-03-22

**Authors:** Yang-Yang Shao, Yi-Ming Zhou, Min Hu, Jin-Ze Li, Cheng-Juan Chen, Yong-Jiang Wang, Xiao-Yun Shi, Wen-Jie Wang, Tian-Tai Zhang

**Affiliations:** 1 State Key Laboratory of Bioactive Substances and Functions of Natural Medicines, Institute of Materia Medica, Chinese Academy of Medical Sciences & Peking Union Medical College, Beijing 100050, China; shaoyangyang@imm.ac.cn (Y.-Y.S.); humin@imm.ac.cn (M.H.); lijz@imm.ac.cn (J.-Z.L.); cchengjuan@imm.ac.cn (C.-J.C.); wwenj@imm.ac.cn (W.-J.W.); 2 Department of Liver disease, Army General Hospital of PLA, Beijing 100700, China; zhzhym2006@163.com; 3 Department of Pharmacy, Changji Vocational and Technical College, Changji 831100, China; 13899670598@163.com; 4 Xinjiang Jinshikang Pharmaceutical Co., Ltd., Urumchi 830000, China; w13909918288@163.com

**Keywords:** allergic rhinitis, anti-allergic activity, Shenqi, degranulation, Th1/Th2 balance

## Abstract

Shenqi is a traditional Chinese polyherbal medicine has been widely used for the treatment of allergic rhinitis (AR). The aim of this study was to investigate the anti-allergic rhinitis activity of Shenqi and explore its underlying molecular mechanism. Ovalbumin (OVA)-induced allergic rhinitis rat model was used to evaluate the anti-allergic rhinitis effect of Shenqi. The effect of Shenqi on IgE-mediated degranulation was measured using rat basophilic leukemia (RBL-2H3) cells. Primary spleen lymphocytes were isolated to investigate the anti-allergic mechanism of Shenqi by detecting the expression of transcription factors via Western blot and the level of cytokines (IL-4 and IFN-γ) via ELISA. In OVA-induced AR rat models, Shenqi relieved the allergic rhinitis symptoms, inhibited the histopathological changes of nasal mucosa, and reduced the levels of IL-4 and IgE. The results from the in vitro study certified that Shenqi inhibited mast cell degranulation. Furthermore, the results of GATA3, T-bet, p-STAT6, and SOCS1 expression and production of IFN-γ and IL-4 demonstrated that Shenqi balanced the ratio of Th1/Th2 (IFN-γ/IL-4) in OVA-stimulated spleen lymphocytes. In conclusion, these results suggest that Shenqi exhibits an obvious anti-allergic effect by suppressing the mast cell-mediated allergic response and by improving the imbalance of Th1/Th2 ratio in allergic rhinitis.

## 1. Introduction

Allergic rhinitis (AR) is a common chronic inflammatory disease, which affects the health of nearly 30% of the world population [1]. It is characterized by inflammation of nasal mucosa with hypersensitivity resulting from all kinds of allergens. Typical symptoms of AR including sneezing, rhinorrhea, nasal rubbing, nasal congestion, and obstruction, seriously affecting patients’ quality of life and work [2,3].

An allergic reaction is an immunological disorder and caused by genetic and environmental factors, among others [4]. Among immune cells, T helper type 2 (Th2) cells and mast cells play crucial roles in the pathogenesis of allergic responses in AR. Activation of Th2 cells by antigens induces a production of Th2 cytokines, such as IL-4, which promotes a special production of immunoglobulin E (IgE) from B lymphocytes [5,6]. Once IgE binds to the high-affinity immunoglobulin Fc epsilon receptor I (FcεR I), a mast cell surface receptor, special IgE responses to antigens are triggered. Subsequently, mast cells are activated by the complex of IgE-FcεR I on the surface of mast cells, resulting in a degranulation response and secreting allergic mediators including inflammatory cytokines, histamine, β-hexosaminidase, chemokines, and arachidonic derivatives, which results in infiltrating inflammatory cells followed by acute or chronic inflammation in nasal and swollen nasal mucosa [4,7].

Current therapeutic drugs towards AR are limited to antihistamines, anti-allergen, anti-leukotriene, and intranasal corticosteroids that can only alleviate allergic symptoms but fail to regulate the allergic reaction and bring adverse effects such as headache, throat irritation, and nasal dryness. Consequently, researching and developing safe and effective therapeutic agents is necessary, and traditional Chinese medicine is an attractive area for its multi-target combination and regulation of allergic reaction pathogenesis [8,9]. Recently, polyherbal medicine exhibits good potential in the treatment of allergic diseases because of its efficacy and safety [10,11]. Shenqi, a traditional Chinese polyherbal medicine, has been successfully prescribed for the clinical treatment of allergic rhinitis by some traditional healers. The main active compounds of the Shenqi formula include *Codonopsis pilosula* polysaccharide, Astragaloside, Baicalein, Imperatorin, Chlorgenic acid, Indirubin, Luteolin, and Methyleugenol. However, no research on Shenqi has been conducted to reveal possible mechanisms of treatment of AR.

Hence, we hypothesized that the anti-allergic rhinitis action of Shenqi might be associated with inhibition, inflammation, and immunoregulation. In the present study, we aimed to evaluate the effect of Shenqi on allergic inflammatory responses in OVA-induced allergic rhinitis rat models and on IgE-induced mast cell degranulation.

## 2. Results

### 2.1. The Effect of Shenqi on the Nasal Symptoms in OVA-Induced Allergic Rhinitis Rats

**Figure 1:**
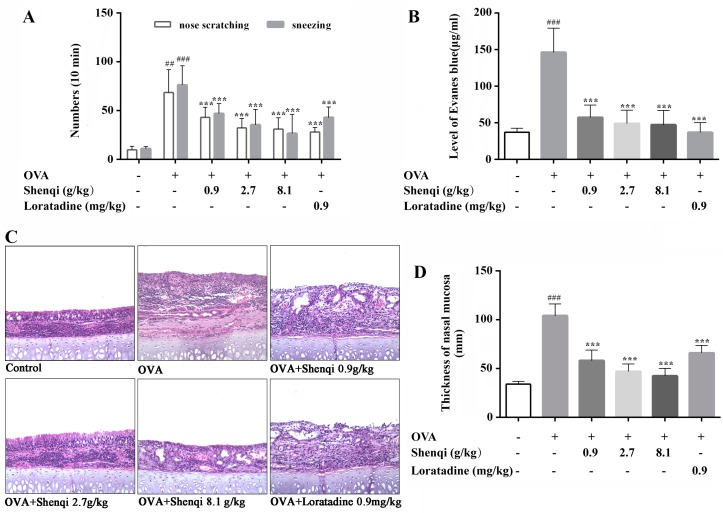
Effects of Shenqi on rhinitis symptoms and histological changes of nasal mucosa in OVA-induced RA models. (**A**) Instances of sneezing and nose scratching for 10 min were used to evaluate the nasal symptoms. (**B**) The permeability of nasal mucosa was detected by perfusing Evans blue in nasal perfusion fluid. Histological changes of nasal mucosa swelling (**C**) and eosinophils lesion (**D**) were observed via microscope after H&E staining (100×). Independent experiments were performed, and the data are presented as the means ± SEM, ^##^ *p* < 0.01 and ^###^ *p* < 0.001 vs. normal rats, *** *p* < 0.001 vs. OVA-treated rats.

To study the anti-allergic effect of Shenqi in vivo, we established the allergic rhinitis rat model and counted instances of sneezing and nose scratching for 10 min after OVA intranasal stimulation on the 30th day of experiment. Our results showed that, after treatment with three doses of Shenqi (0.9, 2.7, and 8.1 g/kg), allergic rhinitis symptoms were significantly alleviated in a dose-dependent manner compared to the untreated control group, The positive agent of Loratadine also displayed an obvious treatment effect on AR rats (Figure 1A).

### 2.2. The Effect of Shenqi on Permeability of Nasal Mucosa

The augment of permeability of nasal mucosa is also a typical characteristic in OVA-induced AR rats. In this study, the permeability of nasal mucosa was measured to evaluate the effect of Shenqi in OVA-induced AR rats by tail vein injection Evans blue. The nasal cavity perfusion fluid was collected to detect the level of Evans blue at various time points, and we then calculated the total Evans blue level of all time points in every experiment group to estimate the permeability of nasal mucosa. As shown in Figure 1B, there was an obvious increase of Evans blue level in OVA-induced rats compared to normal rats. However, the level of Evans blue was significantly decreased in Shenqi-treated groups (0.9, 2.7, and 8.1 g/kg) compared to the untreated OVA-induced model group. The positive agent Loratadine also displayed an obvious preventing effect on the OVA-induced enhanced permeability of nasal mucosa.

### 2.3. The Effect of Shenqi on Histology Changes

In this study, we estimated the effect of Shenqi on the histology changes of nasal mucosa by H&E staining. As shown in Figure 1C, no pathological abnormalities were observed in the nasal mucosa of the normal control group. Conversely, the remarkable mucosa edema, epithelial disruption, and infiltration of eosinophils were observed in the OVA-sensitized group rats in the wall of the nasal cavity. Shenqi at all doses significantly protected the nasal mucosa against lesions compared to the untreated OVA-induced model rats, and obviously decreased the infiltration of eosinophils. Rats treated with Shenqi (0.9, 2.7, and 8.1 g/kg) showed thinner nasal mucosa compared to OVA-sensitized rats, and Loratadine appeared to have a slight inhibitory effect on nasal edema (Figure 1D). The above-mentioned findings indicate that Shenqi prevented nasal destruction and was effective for destructed nasal mucosa recovery.

### 2.4. The Effect of Shenqi on Antigen-Induced Degranulation and Histamine Release in RBL-2H3 Cells

**Figure 2:**
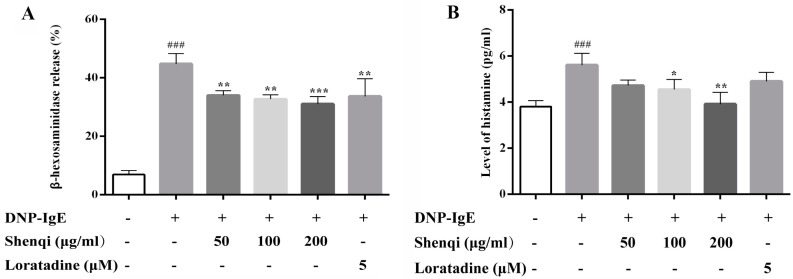
Effects of Shenqi on DNP-IgE-induced RBL-2H3 cell degranulation and the release of histamine. The cells were stimulated with 1 μg/mL DNP-IgE with or without Shenqi, and the levels of β-hexosaminidase (**A**) and histamine (**B**) in supernatants were determined. The data are presented as the means ± SEM of three independent experiments, ^###^ *p* < 0.001 vs. DNP-IgE-free cells, * *p* < 0.05, ** *p* < 0.01, *** *p* < 0.001 vs. DNP-IgE-stimulated cells.

RBL-2H3 mast cells were used to determine the effect of Shenqi on antigen-induced degranulation and histamine release. The β-hexosaminidase activity was measured by assessing the capacity of degranulation in RBL-2H3 cells stimulated with antigen DNP-IgE (1 μg/mL) and challenged with DNP-BSA (200 ng/mL). As shown in Figure 2A, the results indicated that antigen-induced release of β-hexosaminidase was inhibited by Shenqi in a dose-dependent manner, and the decreased level of degranulation capacity reached to 24.10%, 26.99%, and 30.59% at concentrations of 50, 100, and 200 μg/mL, respectively. Antigen DNP-IgE-mediated degranulation led to the increased secretion of histamine compared to normal mast cells, while the release of histamine was significantly inhibited by adding Shenqi (Figure 2B). The inhibitory effect of positive agent Loratadine (5 μM) was relatively weaker than Shenqi at high concentration (200 μg/mL).

### 2.5. The Effect of Shenqi on Cytokines Production and Th1/Th2 Balance

**Figure 3:**
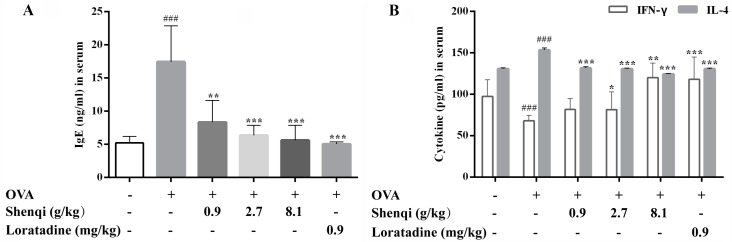
Effects of Shenqi on cytokines production in the serum of OVA-induced AR rats. The level of IgE (**A**) and both IFN-γ and IL-4 (**B**) were detected via ELISA. Independent experiments were performed and the data are presented as the means ± SEM, ^###^ *p* < 0.001 vs. normal rats, * *p* < 0.05, ** *p* < 0.01 and *** *p* < 0.001 vs. OVA-treated rats.

To further analyze whether Shenqi influences the production of mediators that modulate subsequent allergic reaction, the levels of IL-4, IFN-γ, and IgE in the serum or supernatant of lymphocytes were detected via ELISA. After the experiment, the levels of IgE, IL-4, and IFN-γ in the serum were measured to estimate the modulating action of Shenqi on allergic reactions. Our results displayed that Shenqi at doses of 2.7 and 8.1 g/kg significantly decreased the level of IgE caused by OVA stimulation in AR rats (Figure 3A). The content of IL-4 (Th2-related cytokine) and IFN-γ (Th1-related cytokine) in the serum of OVA-induced rats were significantly abnormal than control rats, respectively. Treatment with Shenqi at a dose of 8.1 g/kg obviously decreased the level of IL-4 and increased the level of IFN-γ, compared to those in untreated OVA-induced AR rats (Figure 3B). The imbalance ratio of IFN-γ/IL-4 (Th1/Th2) was thus improved.

**Figure 4:**
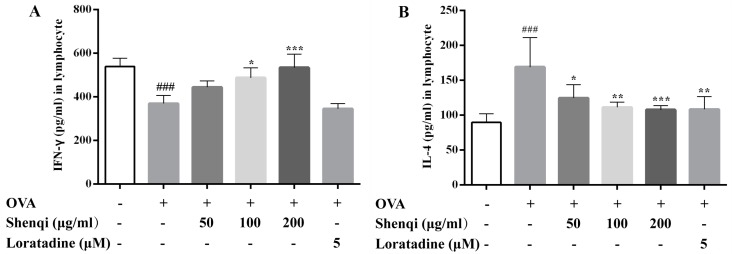
Effects of Shenqi on cytokines production in OVA-stimulated spleen lymphocytes. Primary spleen lymphocytes isolated from Wistar rats were preincubated in the presence or absence of Shenqi (50, 100, and 200 μg/mL) or Loratadine (5 μM) for 1 h and then cultured with OVA for another 72 h, and the supernatants were collected to detect the level of Th1 cytokine IFN-γ (**A**) and Th2 cytokine IL-4 (**B**) via ELISA. The data are presented as the means ± SEM of three independent experiments, ^###^ *p* < 0.001 vs. cells alone, * *p* < 0.05, ** *p* < 0.01 and *** *p* < 0.001 vs. OVA-treated cells.

We further measured the level of cytokines in the cultured supernatant of primary spleen lymphocytes with or without OVA stimulation. As shown in Figures 4A,B, after OVA stimulation, the level of IL-4 was markedly higher, and IFN-γ was lower than control cells and resulted in a significant reduction of ratio of IFN-γ/IL-4 (Th1/Th2). Shenqi significantly increased the level of Th1 cytokine IFN-γ in a dose-dependent manner; otherwise, Shenqi showed an inhibitory effect on the level of IL-4 in a dose-dependent manner. Our results indicated that Shenqi (200 μg/mL) has a stronger regulatory effect on the balance of Th1/Th2 cytokine in lymphocytes than the positive agent Loratadine (5 μM).

### 2.6. Expression of Th1/Th2 Special Transcription Modulating Protein

**Figure 5:**
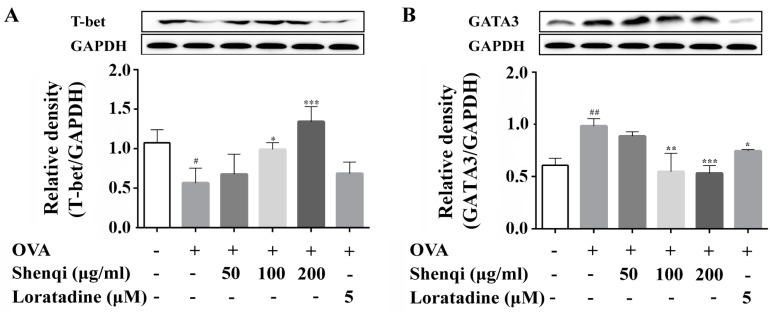

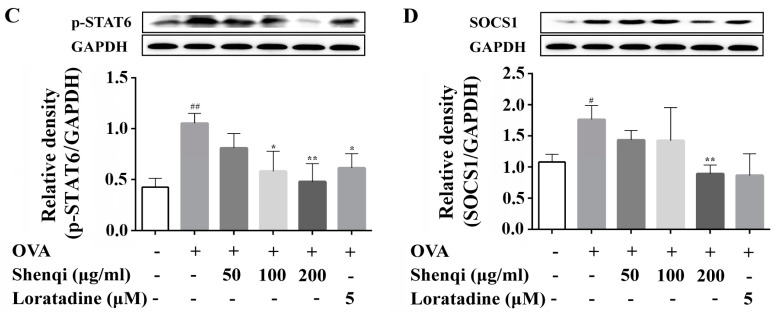
Effects of Shenqi on protein expression of T-bet, GATA3, p-STAT6, and SOCS1 in OVA-stimulated lymphocytes. Primary spleen lymphocytes isolated from Wistar rats were preincubated in the presence or absence of Shenqi (50, 100, and 200 μg/mL) or Loratadine (5 μM) for 1 h and then cultured with OVA for another 72 h. The total cell proteins were extracted to detect the expression of Th1 regulating factor T-bet (**A**); Th2 regulating factor GATA3 (**B**) and phosphor-STAT6 (**C**), as well as the negative regulator SOCS1 (**D**) via Western blot. The data are presented as the means ± SEM of three independent experiments, ^#^ *p* < 0.05, ^##^ *p* < 0.01 vs. cells alone, * *p* < 0.05, ** *p* < 0.01, and *** *p* < 0.001 vs. OVA-treated cells.

To investigate the mechanism by which Shenqi improves the imbalance of IFN-γ/IL-4, the transcription factors of GATA3, T-bet, p-STAT6, modulation protein SOCS1, which are related to the differentiation of Th1 or Th2 and secretion of the IL-4 or IFN-γ, were explored in isolated primary spleen lymphocytes via Western blot. It was found that the expression of GATA3, p-STAT6, and SOCS1 all increased in OVA-sensitized lymphocytes than control cells; on the contrary, the expression of T-bet decreased. Shenqi, especially at a concentration of 200 μg/mL, prevented the increased expression of GATA3, p-STAT6, and SOCS1 and increased the expression of T-bet compared to the untreated OVA-sensitized lymphocytes (Figure 5A–D).

## 3. Discussion

In the present study, we evaluated the anti-allergic rhinitis effect and possible modulating mechanism of Shenqi for the first time, which is an ancient Chinese herbal formula widely used for treating AR. The results demonstrated that Shenqi exhibited remarkable anti-allergic effects by suppressing sneezing, nose scratching, and permeability of nasal mucosa in OVA-induced AR rats. Further investigation implied that the anti-allergic activity of Shenqi is achieved by inhibiting the degranulation in RBL-2H3 mast cells and the production of allergic or inflammatory mediators, such as IgE, histamine, IL-4, and IFN-γ in vivo or in vitro. We also found that the anti-allergic effect of Shenqi was related to improving the imbalance of Th1/Th2 (IFN-γ/IL-4) by modulating the expression of transcription factor GATA3 related to Th2- and T-bet-related to Th1 from primary spleen lymphocytes.

Ovalbumin is widely used to induce AR rat model, this model rat shows the characteristic rhinitis symptoms and pathological changes of AR, such as sneezing, nasal scratching, congestion, and mucosa edema. These are elicited by OVA sensitization and challenge in the in vivo model, resulting in increased levels of histamine and total IgE in the serum and the infiltration of inflammatory cells in the nasal mucosa [12,13]. In our study, OVA-induced AR model rats were used to assess the anti-allergic efficacy of Shenqi. Shenqi treatment remarkably alleviated the AR symptoms, the morphological changes of nasal mucosa, and the IgE production in OVA-sensitized rats. The results demonstrated that Shenqi possessed a treatment effect on AR, and this may be involved in suppressing IgE production in AR model rats.

It is well known that AR is a Type I hypersensitivity allergic reaction mediated through the activity of mast cells by all kinds of allergens and resulting in a degranulation and release of inflammatory mediators, such as histamine, TNF-α, and LTC4 [14]. Therefore, mast cells play a central role in modulating allergic reactions via granulation, and the degranulation of mast cells is considered a key target for anti-allergic drugs. Here, we tried to explore the possible targets of Shenqi in anti-allergic rhinitis. Mast cells were challenged by DNP-IgE to evaluate the efficacy of Shenqi on degranulation. Our study showed that Shenqi significantly inhibited the degranulation and release of histamine in DNP-IgE challenged RBL-2H3 mast cells. It is implied that Shenqi may prevent the activation of mast cells by IgE against the degranulation response.

The infiltration and release of inflammatory mediators by immune cells play a crucial role in the development of allergic disease. In particular, T lymphocytes are key immune cells in allergic responses, especially Th1 and Th2 [15]. T-bet is a Th1 special transcription factor that controls the expression of the hallmark Th1 cytokines, such as IFN-γ [16]. Th1 lymphocytes produce IFN-γ that carries out cell-mediated immunity in auto-immune disease [17]. GATA3 has been shown to induce the Th2 differentiation that depends on STAT6 activation, while suppressing the differentiation towards Th1 cells [18,19]. Th2 cells are characterized to secrete IL-4, which induces an IgE antibody production by B cells to contribute to the development of allergic responses [20]. The Th1 or Th2 subsets also cross-regulate each other, so the balance between IFN-γ/IL-4 cytokines can determine whether the immune response is appropriate or will terminate in detrimental immunopathologies [21,22]. Here, we measured the expression of transcription factors of GATA3 and T-bet, and the levels of IFN-γ and IL-4 in the presence or absence of Shenqi in OVA-induced primary spleen lymphocytes, to investigate the regulatory effect of Shenqi on T cell differentiation. Our findings of the present study showed that the ratio of IFN-γ/IL-4 was obviously reduced in OVA-stimulated primary lymphocytes, and the imbalance was considered towards Th2 (dominant by IL-4) with a reduction of Th1/Th2. However, we demonstrated that Shenqi directly improved the imbalanced ratio of IFN-γ/IL-4 by inhibiting the production of IL-4. The reduction of IL-4 was due to the decreased expression of Th2-specific transcription factor GATA3 and p-STAT6, or the increased expression of Th1-specific transcription factor T-bet. In addition, an upregulated SOCS1 expression correlated with downregulated T-bet and higher expression levels of GATA3 and IL-4 [23]. Our study also showed that Shenqi treatment prevented the higher expression of SOCS1 in OVA-stimulated lymphocytes compared with OVA-stimulated lymphocytes not treated with Shenqi.

## 4. Materials and Methods

### 4.1. Cells and Reagents

RBL-2H3 cell was gifted by Professor Shigang Li (China Three Gorges University). The cells were cultured in Dulbecco’s modification of Eagle’s medium Dulbecco (DMEM, Invitrogen, NY, USA) containing 10% fetal bovine serum (FBS, Invitrogen, Carlsbad, NY, USA), at 37 °C with 5% CO_2_ in a humidified atmosphere. Primary spleen lymphocytes were isolated from Wistar rat and cultured in RPMI Medium 1640 (Invitrogen, Carlsbad, NY, USA) with 10% FBS, 100 U/mL penicillin (Gibco, Grand Island, NY, USA), and 100 μg/mL streptomycin (Gibco, Grand Island, NY, USA). Ovalbumin (OVA grade V), 4-niroPheny 1-*N*-acety-β-d-glucosaminide, and anti-dinitrophenyl IgE antibody (anti-DNP IgE) were purchased from Sigma-Aldrich Co (St. Louis, MO, USA), and DNP-bovine serum albumin (BSA) was purchased from Invitrogen (Carlsbad, NY, USA). The following primary antibodies of GATA3 (ab99428), T-bet (ab91103), p-STAT6 (Tyr641, ab54461), and SOCS1 (ab62584) were purchased from Abcam (Cambridge, MA, USA). Evans blue was purchased from Tokyo chemical industry Co., Ltd. (Tokyo, Japan), and histamine and IgE ELISA kits were purchased from Shanghai Jianglai industrial Limited By Share Ltd. (Shanghai, China). IL-4 and IFN-γ ELISA kits were purchased from Hangzhou Cluster Technology Limited Biotechnology Co., Ltd. (Hangzhou, China). Loratadine was purchased from Shanghai Schering-Plough Pharmaceutical Co., Ltd. (Shanghai, China). Shenqi was provided by Xinjiang Jin Shi Kang Pharmaceutical Co., LTD. (Urumchi, China).

### 4.2. Preparation of Shenqi

The herb formula Shenqi consisted of 10 different traditional Chinese herbs, including the following: 20 g of *Angelicae dahuricae* radix; 10 g each of *Scutellariae* radix, *Lonicerae japonicae* flos, *Menthae haplocalycis* herrrba, *Astragali* radix, and *Codonopsis* radix; 30 g each of *Astragali* radix, *Codonopsis* radix, and *Agrimoniae* herba; and 5 g of *Asari* radix et rhizome (Table 1). The voucher specimens of the 10 herbs were deposited at Herbarium, Institute of Materia Medica, Chinese Academy of Medical Sciences, Beijing, China, with voucher numbers as follows: *Angelica dahurica* (Fisch. exHoffm.) Benth. et Hook. f., ID-S-600; *Scutellaria baicalensis* Georgi, ID-S-2523; *Lonicera japonica* Thunb., ID-S-1107; *Mentha haplocalyx* Briq., ID-S-1747; *Astragalus membranaceus* (Fisch.) Bunge, ID-S-2173; *Codonopsis tangshen* Oliv., ID-S-480; *Isatis indigotica* Fort., ID-S-2084; *Taraxacum mongolicum* Hand.–Mazz., ID-S-2528; *Agrimonia pilosa* Ledeb., ID-S-1086; *Asarum sieboldii* Miq., ID-S-1193.

Shenqi is manufactured by Xinjiang Jinshikang Pharmaceutical Co., Ltd. (Urumchi, China). Briefly, each herb was sliced to an appropriate size. The mixture of *Asari* radix, rhizome, and *Menthae haplocalycis* herrrba was extracted in distilled water (1:8, *w/v*) for 5 h, and volatile oil was collected and formed a clathrate compound to be used. The mixture of *Scutellariae* radix, *Lonicerae japonicae* flos, *Codonopsis tangshen* Oliv, *Taraxaci* herba, and *Agrimoniae* herba was decocted in boiling distilled water (1:8, *w/v*) for 1 h three times. *Angelicae dahuricae* radix, *Isatidis* folium, and *Astragali* radix were mixed to extract in 75% ethanol (1:8, *w/v*) for 1 h twice. Above liquid extraction was filtered using a 100 mesh filter and condensed under the reduced pressure to obtain concentrations of 1.20–1.25 relative density, respectively. Concentrations were combined and vacuum dried at 60 °C to yield a powder, and the powder was then mixed with the clathrate compound and dried at 50 °C to yield a new powder. One gram of yielded powder was equivalent to 1.9 g of the total raw herbs.

### 4.3. Animals

A total of 108 male Wistar rats (180–200 g) was purchased from Beijing Wei Tong Li Hua experimental animal technical Co., Ltd. (Beijing, China). Rats were maintained in the Animal Resource Facility. They were fed standard laboratory chow and water ad libitum, and kept under a 12 h dark/light cycle. The animal experiment was conducted with an approval form and under the supervision of the Laboratory Animal Welfare and Ethics Committee of the Institute of Materia Medica, Chinese Academy of Medical Sciences (No. 571/2016). Animal surgeries were performed under sodium pentobarbital anesthesia with minimal suffering.

### 4.4. OVA-Induced AR Rat Models and Shenqi Treatment

Rats were sensitized by OVA as follows. OVA (0.3 mg/mL) diluted in aluminum hydroxide gel and was used to sensitize rats seven times via intraperitoneal injection on Days 1, 3, 5, 7, 9, 11, and 13. Then, sensitized rats were intranasally challenged by daily dropping with OVA diluted with sterile physiological saline (20 μL/nostril, 50 mg/mL) from Days 15 to 21 [24].

As the OVA-induced allergic rhinitis model was successfully established, rats were randomly divided into six groups (*n* = 18 per group) that received different agents by daily intragastric administration for 7 days. There was a control group and a model group with distilled water, three Shenqi treatment groups at doses of 0.9, 2.7, and 8.1 g/kg, respectively, and a positive agent group with Loratadine at a dose of 0.9 mg/kg.

### 4.5. The Effects of Shenqi on OVA-Induced Rats

#### 4.5.1. Evaluation of Nasal Symptoms

On the 30th day of the experiment, rats were stimulated by OVA (20 μL/nostril, 50 mg/mL), dissolved in physiological saline solution into the bilateral nasal cavities, and then placed into the observation cage. Instances of sneezing and nose scratching were counted for 10 min after the OVA intranasal stimulation to evaluate the degree of allergic responses. Rats in the normal control were processed with saline.

#### 4.5.2. Permeability of Nasal Mucosa

The permeability of nasal mucosa was detected to evaluate the protection effect of Shenqi on OVA-induced impairment of rat nasal mucosa. Briefly, rats were anesthetized with 10% chloral hydrate (0.3 mL/100 g), and 1% Evans blue (1 mL) was then injected in the tail vein. Tracheotomy was performed and perfused with preheated (37 °C) 1% OVA into a nasal cavity by peristaltic pump at a speed of 0.2 mL/min. Perfusion fluid was collected every 15 min to 120 min. Collected perfusion fluid samples were centrifuged at 3000 *g* for 15 min, and for 100 μL of each sample, supernatants were obtained to detect the absorbance at 610 nm using a microplate reader. The permeability of nasal mucosa was evaluated by calculating the total level of Evans blue in perfusion fluid. 

#### 4.5.3. Histological Analysis of Nasal Mucosa

After the experiment, rats were sacrificed by intraperitoneal overdose of chloral hydrate, and the nasal mucosa was separated and fixed in 4% neutral buffered formalin at room temperature. After fixation, tissues were then paraffin embedded, sectioned, and stained with hematoxylin and eosin (H&E) to examine eosinophils lesions and nasal mucosa swelling.

### 4.6. Degranulation and Histamine Release Assay for RBL-2H3 Cells

Degranulation was estimated by detecting β-hexosaminidase release according to the reference of Wang et al. [25]. Briefly, RBL-2H3 cells were seeded in 96-well plates at a density of 2.5 × 104 cells/well and sensitized overnight with mouse monoclonal anti-dinitrophenyl immunoglobulin E (DNP-IgE) antibody (1 μg/mL). The cells were washed twice with Tyrode’s solution (137 mM NaCl, 2.7 mM KCl, 0.4 mM NaH_2_PO_4_, 1 mM MgCl_2_, 12 mM NaHCO_3_, and 1.8 mM CaCl_2_, hepes 20 mM, BSA 1 mg/mL, pH 7.4). Then, the cells were treated with Shenqi (50, 100, and 200 μg/mL) or Loratadine (5 μM, positive control) at 37 °C for 1 h, and antigen dinitrophenyl-bovine serum albumin (DNP-BSA) (200 ng/mL) was then added to stimulate for another 1 h at 37 °C to induce degranulation. The reaction was stopped by putting the plate in an ice bath. Fifty microliters of supernatant was collected to measure the level of histamine via an ELISA kit according to the manufacturer’s instructions.

To determine the β-hexosaminidase release, 50 μL of supernatant was incubated with 50 μL of substrate (1 mM *p*-nitrophenly-*N*-acetyl-β-d-glucosaminide in 0.1 M citrate buffer, pH 5.0) in 96-well plates at 37 °C for 1 h. The reaction liquid was collected to measure the concentration of β-hexosaminidase in supernatant. The total β-hexosaminidase activity in the cells was also detected. Cells were lysed with an equal volume of 0.1% Triton-X 100 at 37 °C for 1 h. The reaction was terminated by adding 150 μL of 0.1 M carbonate buffer (Na_2_CO_3_/NaHCO_3_, pH 10.0). Lysates were collected to measure the concentration of β-hexosaminidase as total level. The absorbance was measured at 405 nm by a microplate reader (Synergy H1, BioTeck, VT, USA). The β-hexosaminidase release rate was calculated by the following formula:

β-hexosaminidase release (%) = (ODsupernatant − ODbasal)/(ODtotal − ODbasal).

### 4.7. Cytokines Measurement

After the animal experiment, AR rats were sacrificed, and blood samples were collected. All blood samples were centrifuged at 3000 *g* for 15 min at 4 °C. Serum was collected and conserved at −20 °C. The levels of cytokines IL-4, IFN-γ, and IgE were measured via ELISA.

Primary rat spleen lymphocytes were obtained from normal Wistar rats by isolation kit (Tianjin Hao Yang biological manufacture Co., Ltd., Tianjin, China) according to the manufacturer’s instructions. The isolated primary cells (6 × 10^6^ cells/mL) were seeded in 6-well plates overnight and pretreated with Shenqi (50, 100, and 200 μg/mL) or Loratadine (5 μM) at 37 °C for 1 h, and the cells were then stimulated with OVA (200 μg/mL) for another 72 h. The concentration of IL-4 and IFN-γ in the cell culture supernatants were measured using ELISA kits according to the manufacturer’s instruments. The cells were prepared to detect the protein expression via Western blot.

### 4.8. Western Blot

Primary spleen lymphocytes treated with Shenqi and OVA as above described were harvested and resuspended in lysis buffer with RIPA (Beyotime, Shanghai, China) on ice for 30 min to obtain total protein. Concentration of protein was detected with a BCA protein assay kit (Thermo, Rockford, IL, USA) and loading buffer was added to the sample. The sample was then heated at 100 °C for 5 min. Equal amounts of protein from the different samples were separated by 10% sodium dodecyl sulfate poly-acrylamide gel electrophoresis (SDS-PAGE), and then transferred to PVDF membranes. After being blocked with 5% nonfat milk for 1 h, the PVDF membranes were incubated overnight at 4 °C with the primary antibodies that recognized GATA3, T-bet, p-STAT6, and SOCS1. Anti-rabbit horseradish-linked IgG was used as the secondary antibody. Signals were detected using electrochemiluminescence (ECL, Millipore, MA, USA).

### 4.9. Statistical Analysis

Data are presented as means ± SEM. unless otherwise indicated. The statistical significance of difference among multiple groups was analyzed by a one-way ANOVA—Ordinary (GraphPad software, Prism 5.0, GraphPad software Inc., San Diego, CA, USA). The significance of differences between the normal and control groups was evaluated using Student’s *t*-tests. *p* < 0.05 was considered significant.

## 5. Conclusions

The present study showed that Shenqi, as a traditional Chinese herbal formula, exerted significant anti-allergic effects by regulating the differentiation of T lymphocytes and inhibiting the production of allergic and inflammatory mediators both in vivo and in vitro. These findings confirm the efficacy of Shenqi on anti-allergic disease, especially allergic rhinitis. Meanwhile, our studies provide an underlying mechanistic evidence for the wide application of Shenqi in the treatment of allergic rhinitis.

## Figures and Tables

**Table 1:** The herbal prescription of Shenqi ^a^.

Herbal Name	Plant Part	Ratio of Each Plant in the Formula (%)
*Angelica dahurica* (Fisch. exHoffm.) Benth. et Hook. f.	Root	12.12
*Scutellaria baicalensis* Georgi	Root	6.06
*Lonicera japonica* Thunb.	Flower	6.0610
*Mentha haplocalyx* Briq.	Ground part	6.06
*Astragalus membranaceus* (Fisch.) Bunge	Root	18.18
*Codonopsis tangshen* Oliv.	Root	18.18
*Isatis indigotica* Fort.	Leaf	6.06
*Taraxacum mongolicum* Hand.–Mazz.	Herba	6.06
*Agrimonia pilosa* Ledeb.	Groud part	18.18
*Asarum sieboldii* Miq	Root	3.04

^a^ The prescription is from Chinese Pharmacopoeia 2015.
